# The ROPScore as a Screening Algorithm for Predicting Retinopathy of Prematurity in a Brazilian Population

**DOI:** 10.6061/clinics/2018/e377

**Published:** 2018-07-23

**Authors:** Kellen Cristiane do Vale Lucio, Maria Regina Bentlin, Ana Carolina de Lima Augusto, José Eduardo Corrente, Taísa Bertoco Carregal Toscano, Regina El Dib, Eliane Chaves Jorge

**Affiliations:** Universidade Estadual Paulista Julio de Mesquita Filho (UNESP), Botucatu, SP, BR

**Keywords:** Retinopathy of Prematurity, Preterm Infants, Algorithm

## Abstract

**OBJECTIVES:**

To evaluate the accuracy of the ROPScore algorithm as a predictor of retinopathy of prematurity (ROP).

**METHODS:**

A prospective cohort of 220 preterm infants with a birth weight ≤1500 g and/or gestational age ≤32 weeks was included. The ROPScore was determined in the sixth week of life in 181 infants who then survived until a corrected gestational age of 45 weeks. The sensitivity, specificity, and positive (PPV) and negative predictive values (NPV) of the algorithm were analyzed.

**RESULTS:**

ROP was found in 17.6% of the preterm infants. The sensitivity of this test for any stage of ROP was 87.5%, while that for severe ROP was 95.4% (21/22 cases). The PPV and NPV were 59.6% and 97%, respectively, for any stage of ROP and 44.7% and 99.25%, respectively, for severe ROP. The ROPScore could therefore hypothetically reduce the number of ophthalmologic examinations required to detect ROP by 71.8%.

**CONCLUSION:**

The ROPScore is a useful screening tool for ROP and may optimize examinations and especially the identification of severe ROP.

## INTRODUCTION

In recent years, substantial improvements in neonatal care have increased survival in preterm infants with very low birth weight 1. They have also increased the incidence of retinopathy of prematurity (ROP), a disease of the developing retinal vasculature that is the leading cause of preventable childhood blindness worldwide, especially in developing countries 2. Timely detection of ROP is very important to protect visual functions in preterm infants 3. The most important risk factors for ROP are birth weight (BW) and gestational age (GA); however, others factors, including the length of oxygen-therapy, sepsis, blood transfusion, bronchopulmonary dysplasia, and hyperglycemia, are also associated with postnatal morbidities 4,5. Several more recent studies have identified poor postnatal weigh gain as a strong predictor of ROP 6-8.

Many screening guidelines based on BW and GA have been proposed to identify preterm infants at risk of developing severe ROP. These guidelines have been adapted to account for difference in population characteristics in several countries 9-12. The Brazilian guidelines for screening and treating ROP recommend that all the preterm infants with GA ≤32 weeks and BW ≤1500 g should be screened by fundus examination starting between the fourth and sixth weeks of life until complete retinal vascularization is achieved 13.

Timely screening is costly and requires a large amount of work by health professionals 14. Furthermore, the excessive number of examinations currently required to identify which preterm infants need treatment can lead to stress and cardiorespiratory instability in patients with other comorbidities 15,16. The need to optimize screening so that efforts can be directed to preterm infants at higher risk of ROP and the burden of examinations on neonates can be reduced has led to the development of predictive algorithms that use BW and GA in addition to weight gain to measure postnatal growth 17.

The ROPScore is a simple scoring system that was proposed by Eckert et al. 14 to predict severe ROP. This algorithm uses BW, GA, proportional weight gain at the sixth week of life, history of blood transfusion, and use of oxygen in mechanical ventilation as predictive variables.

The score is calculated by an Excel spreadsheet once per infant in the sixth week of life. High score values indicate a high risk of developing severe ROP.

Only one study has validated this screening tool. That study used a retrospective design to analyze an Italian population of 445 preterm infants 9. The purpose of the present study was to evaluate the efficacy of the ROPScore as a method for predicting severe ROP in a population of preterm infants in Brazil.

## METHODS

We performed a prospective cohort study in which we included all preterm infants born with BW ≤1500 g and GA ≤32 weeks who were admitted to the Neonatal Intensive Care Unit (NICU) of the University Hospital of Botucatu Medical School - UNESP, Brazil, from November 2012 to July 2014. The study protocol was approved by the Research Ethics Committee of the Botucatu Medical School – UNESP (no. 4051/2011), and the parents/guardians of all included infants provided written consent to participate in the study. Infants that died before completing six weeks of life or before reaching 45th weeks of corrected gestational age. No other exclusion criterion was used. ROP screening was performed between the fourth and sixth weeks after birth and repeated based on the findings of ophthalmologic examinations performed at intervals determined by the Brazilian guidelines for detecting and treating ROP, which state that exams should be performed until the retina is fully vascularized or ROP has totally regressed 13. Ophthalmologic examinations consisted of binocular indirect ophthalmoscopy after pupillary dilatation with tropicamide 0.5% and phenylephrine 2.5% and were performed using a 28-diopter lens and an eye speculum. ROP was categorized according to the International Classification of Retinopathy of Prematurity Revised 18. Clinical outcomes were defined as the onset of any stage of ROP (requiring no treatment) or severe ROP that required treatment. Each child was classified according to the most advanced ROP stage observed. The indications for treatment were based on the Early Treatment for Retinopathy of Prematurity study (ETROP) criteria 19.

**Figure 1:**
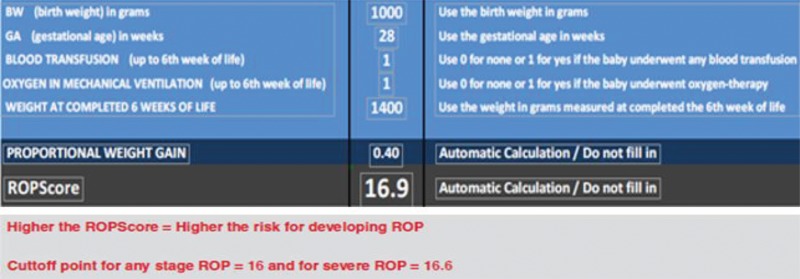
Excel spreadsheet (Microsoft) used to calculate the ROPScore. From Eckert et al. 2012.

ROPScore Screening was applied in the sixth week of life using a Microsoft® Excel spreadsheet as proposed by Eckert et al. 14. This process required the following parameters: BW, GA, weight at the sixth week of live, the presence or absence of blood transfusion up to the sixth week of life, and oxygen in mechanical ventilation (Figure 1).

## RESULTS

A total of 220 preterm infants met the inclusion criteria, thirty-eight of whom died before the sixth week of life. Thus, 181 patients (86 male and 95 female) completed the study. The prevalence of any stage of ROP was 32/181 infants (17.6%). Ten preterm infants developed low-grade ROP, and 22/181 developed severe ROP that required treatment (12.1%). The baseline demographics and clinical characteristics for this cohort are shown in Table 1.

**Figure 2:**
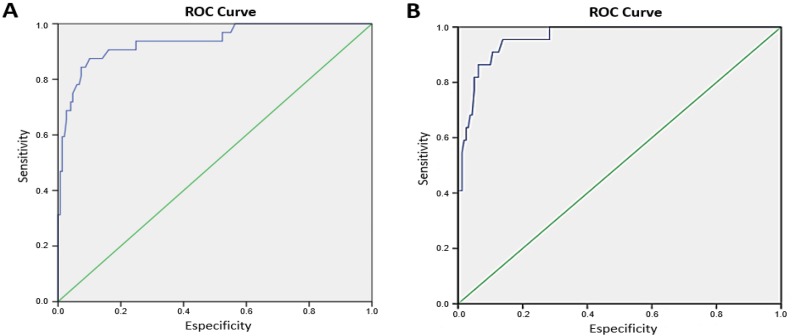
Receiver operating characteristic (ROC) curves for the detection of any stage of retinopathy of prematurity (ROP) (A) and of severe ROP (B) according to the ROPScore algorithm.

The accuracy of the ROPScore for predicting the onset of ROP in our population was determined by ROC curves (Figure 2), and cut-off points for sensitivity and specificity were obtained for continuous score values. The ROPScore values ranged from 7.2 to 19.6 (Table 1). The best cut-off point established for any stage of ROP was 16 (87.5% sensitivity and 87.2% specificity), while that for severe ROP was 16.6 (95.4 sensitivity and 83.6% specificity). The positive and negative predictive values (PPV and NPV, respectively) for any stage of ROP and severe ROP are shown in Table 2.

## DISCUSSION

In Brazil, the prevalence of ROP varies according to region, the level of neonatal care, and access to ophthalmologic screening programs. The blindness caused by ROP can be prevented with timely screening 20. In the present study, the ROPScore was a useful and accurate method for predicting ROP.

Scoring systems have become widely used in neonatology, including neonatal intensive care, to help detect comorbidities. Predictive algorithms represent promising and appropriate tools that can be used to identify preterm infants at risk of developing severe ROP and reduce the excessive number of examinations performed per preterm infant 21.

The ROPScore was developed in Brazil 14 and was chosen to be tested in our population because it is simple and practical to use and requires only one weight measurement.

The incidence of severe ROP was much higher in this sample than in the population studied by Eckert et al. 14 in South Brazil (12.5% *versus* 5%, respectively). A comparison of the characteristics of that population *versus* the those of the present cohort revealed that in preterm infants who developed severe ROP, BW (908.7 g±232.6 *versus* 763.1±186.8), GA (27.9±2.2 *versus* 25.9±1.2) and weight gain during the first six weeks of life (411.7±277.4 *versus* 390.7±162.8) were lower in this study than in the previous study, and this may account for the fact that more infants developed the more severe form of the disease in this study.

The cut-off point for ROPScore was higher in this cohort (16 for any stage of ROP and 16.6 for severe ROP) than in the study population in Eckert et al. 14 (11 for any stage of ROP and 14.5 for severe ROP). Piemarocchi et al. 9 evaluated ROPScore in a retrospective cohort but adjusted only the cut-off point for severe ROP, which increased from 14.5 to 15.8.

The NPV calculated in this study indicated that the probability that a preterm infant with a ROPScore below a cut-off point of 16 would not develop any stage of ROP was 97.1%, while the probability that the same infant would not develop the severe form of the disease was 99.2%. An adjusted ROPScore correctly identified 28 of 32 preterm infants who developed any stage of ROP and 21 of 22 who developed severe ROP. Despite the fact that one case of severe ROP was not identified, the adjusted ROPScore had a high NPV and was associated with high sensitivity, indicating that it was a useful tool for identifying preterm infants at greater risk and would, therefore, reduce the number of exams in clinical practice. If the ROPScore were applied, 130 of the preterm infants in this cohort would not need to be evaluated with the same frequency, resulting in a decrease of 71.8% in the total number of tests needed to detect ROP.

The introduction of such algorithms is still in the preliminary phase, and it should be stated that the goal is not to replace the current screening guidelines. However, these tools can help to reduce the number of lost diagnoses in ROP 7,9. Regardless of this positive characteristic of the function of the algorithms, there are limitations to their clinical use. First, the ROPScore calculation uses preterm weight only at the sixth week of life. Hence, this test may not detect some high-risk preterm infants in whom aggressive posterior ROP is initiated prior to weight measurement but then subsequently evolves rapidly 9. Moreover, the early hospital discharge of preterm infants that are evolving well is another factor that contributes to the loss of a weighing on the correct day and the consequent inability to apply the ROPScore.

Accordingly, other predictive models that are currently being tested in ROP in addition to the ROPScore also have limitations. For example, WINROP 2 22 was proposed for European populations and has been validated by several studies that have shown it has good effectiveness in predicting ROP. However, some studies have shown that this score does not perform well in underdeveloped countries in which moderate and late preterm infants can also develop ROP 23-26. These include a study by Ko et al. 24 in which the authors concluded that WINROP was especially effective in preterm infants with BW <1000 g or GA <28 weeks and did not detect six neonates with severe ROP. In Brazil, Hard et al. 25, also reported that some cases with severe ROP were lost when they used WINROP, and they suggested that the algorithm needed to be reformulated with data from developing countries.

CHOP-ROP is another simple model. However, it limits GA to <30 weeks and requires daily weighing, which restricts its usefulness in clinical practice 26. A separate model, the CO-ROP, was recently proposed and is still being validated 27.

In conclusion, we demonstrate that the ROPScore was an effective, promising, and noninvasive screening tool for predicting ROP in a Brazilian population of preterm infants. The results of Eckert et al. 14 are compatible with those obtained in this cohort with regard for a high score for sensitivity and a high VPN. With regard for ROPScore cut-off points, although we adjusted the values for our population (16 and 16.6, for any stage and severe ROP), the cut-off values used in the original cohort 14 would have been sufficient to detect all preterm infants with severe ROP.

Although the introduction of algorithms such as the ROPScore is still in the preliminary phase, and the goal of such algorithms is not to replace the current screening guidelines, they can help to reduce the number of lost ROP diagnoses. With regard for this function of the algorithms, one difficulty we encountered in using the ROPScore was that it required assessing preterm weight only in the sixth week of life. Some high-risk preterm infants in whom aggressive ROP initiates prior to this weight measurement can evolve rapidly and may not be detected 9. Additionally, the early pre-term hospital discharge of infants that are evolving well is another factor that contributes to the loss of a correct weigh time and therefore the loss of the ROPScore.

Finally, the process by which a scoring system is validated is a dynamic one. In the current study, we aimed to contribute by validating the real-world usefulness of the ROPScore in Brazil. New prospective studies are needed to determine the impact of the ROPScore in a clinical setting in different populations.

## AUTHOR CONTRIBUTIONS

El Dib R, Lucio KC and Jorge EC conceived and designed the study, drafted the initial manuscript, and reviewed and revised the manuscript. Toscano TB, Augusto AC, Lucio KC and Corrente JE collected data, carried out the initial analyses, and reviewed and revised the manuscript. Jorge EC, El Dib R and Bentlin MR designed the data collection instruments, coordinated and supervised data collection, and critically reviewed the manuscript. All authors approved the final manuscript as submitted and agree to be accountable for all aspects of the work.

## Figures and Tables

**Table 1:** Characteristics of the 181 premature infants included in the study.

Characteristic	Total Cohort	Any stage ROP	Severe ROP
**Number of patients**	181	32	22
**Male**	86/181	10/32	6/22
Mean BW (g)*	1271.6 ± 354.6	884.0 ± 250.0	763.1±186.8
Mean GA (weeks)*	29.2 ± 2.2	26.4±1.6	25.9±1.2
Mean WG at the sixth week of life (g)*	596.9 ±248.0	407.4±190.8	390.7±162.8
ROPScore range*	7.2 – 19.6 (13.5±3.0)	12 – 19.6 (16.0±2.3)	14.7 – 19.6 (17.9±1.0)

* Data are expressed as the mean ± SD; BW: Birth Weight; GA: Gestational Age; ROP: Retinopathy Of Prematurity; SD: Standard Deviation; WG: Weight Gain from birth to 6 weeks of life.

**Table 2:** Accuracy of the ROPScore for predicting the development of ROP.

	Any stage ROP	Severe ROP
	ROPScore: ≥16	ROPScore: ≥16.6
Sensitivity	87.5% (76%-98.9%)	95.4% (86.7%-100%)
Specificity	87.2% (81.9%-92.6%)	83.6% (77.9%-89.4%)
PPV	59.5% (45.5%-73.6%)	44.7% (30.5%-58.9%)
NPV	97.1% (94.1%-99.9%)	99.2% (97.8%-100%)

NPV: Negative Predictive Value; PPV: Positive Predictive Value; ROP: Retinopathy Of Prematurity.
